# Protected carotid artery stenting in patients with severe stenosis

**DOI:** 10.1097/MD.0000000000030106

**Published:** 2022-08-19

**Authors:** Seo-Young Jeon, Jong-Myong Lee

**Affiliations:** a Jeonbuk National University Hospital & Medical School, Jeon-Ju, Republic of Korea; b Department of Neurosurgery, Jeonbuk National University Hospital & Medical School, Jeon-Ju, Republic of Korea.

**Keywords:** carotid artery, intraplaque hemorrhage, stent, ulcer

## Abstract

Intraplaque hemorrhage (IPH) and ulcers are the major findings of unstable plaques. In addition, initial symptoms are associated with postprocedural complications after carotid artery stenting (CAS). The aim of this study was to determine the safety of CAS using an embolic protection device in symptomatic patients with severe carotid artery stenosis and unstable plaques such as IPH and ulcers.

This retrospective study included 140 consecutive patients with severe carotid stenosis. These patients underwent preprocedural carotid vessel wall imaging to evaluate the plaque status. We analyzed the incidence of initial clinical symptoms, such as headache, nausea, and vomiting, after CAS. The primary outcomes analyzed were the incidence of stroke, myocardial infarction, and death within 30 days of CAS.

Sixty-seven patients (47.9%) had IPH, and 53 (38.9%) had ulcers on carotid wall imaging/angiography. Sixty-three patients (45.0%) had acute neurological symptoms with positive diffusion-weighted image findings. Intraluminal thrombi on initial angiography and flow arrest during CAS were significantly higher in patients with IPH and symptomatic patients. Symptoms were significantly higher in patients with IPH than in those without (63.5% vs 35.1%, *P* < .001). There were no significant differences in clinical symptoms after stenting or in primary outcomes, regardless of IPH, ulcer, or initial symptoms.

IPH and plaque ulceration are risk factors in symptomatic carotid stenosis. However, IPH and plaque ulceration were not a significant risk factors for cerebral embolism during protected carotid artery stent placement in patients with carotid stenosis. Protected CAS might be feasible and safe despite the presence of unstable plaques.

## 1. Introduction

Atherosclerotic carotid artery stenosis accounts for approximately 20% of all ischemic stroke cases.^[1]^ Some characteristics such as intraplaque hemorrhage (IPH), ulcers, calcification, thin fibrous cap, and lipid-rich necrosis core are associated with vulnerability to atherosclerosis. High-resolution magnetic resonance imaging (HR-MRI) enables the assessment of plaque composition, which contributes to plaque vulnerability.^[2–4]^ With the progress of this technique, features of unstable plaques can be detected on imaging. These features are strongly associated with the occurrence of clinical cerebrovascular events.^[5]^ Moreover, they could be influential predictors of ischemic stroke.^[6,7]^

There are 2 main therapeutic strategies for carotid artery stenosis: carotid endarterectomy and carotid artery stenting (CAS). Carotid endarterectomy is believed to be a radical and effective method for the treatment of carotid artery stenosis.^[8]^ Studies have shown that it is effective in both symptomatic and asymptomatic patients. Recently, CAS has been suggested as a new method for the treatment of carotid artery stenosis. It has been established as an option for patients at a high surgical risk. Long-term functional outcome and risk of fatal or disabling stroke are similar for stenting and endarterectomy for symptomatic carotid stenosis.^[9–12]^

There is ongoing debate on whether the presence of an unstable plaque is a risk factor for complications after CAS. Yoshimura et al^[13]^ reported that high-intensity signal on time-of-flight (TOF) MR angiography indicated a high risk of cerebral embolism during CAS. However, Yoon et al^[14]^ suggested that protected CAS was safe in patients with severe carotid stenosis and IPH. In a previous prospective study from our group, we reported that protected CAS appeared to be safe, regardless of the noted unstable plaque findings as seen on carotid MRI. In addition, the relationship between unstable plaques on carotid HR-MRI and the safety of CAS has not been clarified. The role of carotid HR-MRI in the selection of patients with CAS remains unclear.

This retrospective study aimed to determine the safety of CAS using an embolic protection device in symptomatic patients with severe carotid artery stenosis and unstable plaques such as IPH and ulcers.

## 2. Materials and Methods

The study protocol was approved by the institutional review board. The requirement of informed consent from the relatives of the decrease was waived.

### 2.1. Patients

This retrospective study assessed 175 consecutive patients with severe carotid stenosis treated between January 2014 and October 2018. According to the North American Symptomatic Carotid Endarterectomy Trial criteria, severe stenosis is defined as stenosis >70%.^[15]^ We initially performed carotid sonography and routine brain MRI including MR angiography for the diagnosis of carotid artery stenosis and selection of carotid plaque MRI. Carotid plaque MRI was performed within 3 days before CAS. Of the 175 consecutive patients who underwent carotid MRI and CAS, 21 were excluded because of incomplete coverage of the carotid artery bifurcation (n = 11) or poor imaging quality (n = 10). Fourteen patients were excluded because of additional stenotic lesions in the territorial intracranial artery (cavernous or petrous internal carotid artery [ICA], 7; middle cerebral artery, 7). The inclusion criteria were given as follows: symptomatic and/or asymptomatic stenosis in the ICA or carotid bifurcation; CAS procedures with cerebral protection through the common femoral artery; and number of periprocedural complications such as stroke, death, or myocardial infarction (MI) within 30 days reported separately. Stroke was defined as any sudden neurological deficit due to cerebral infarction.

### 2.2. Carotid plaque MR imaging

**Figure 1.**
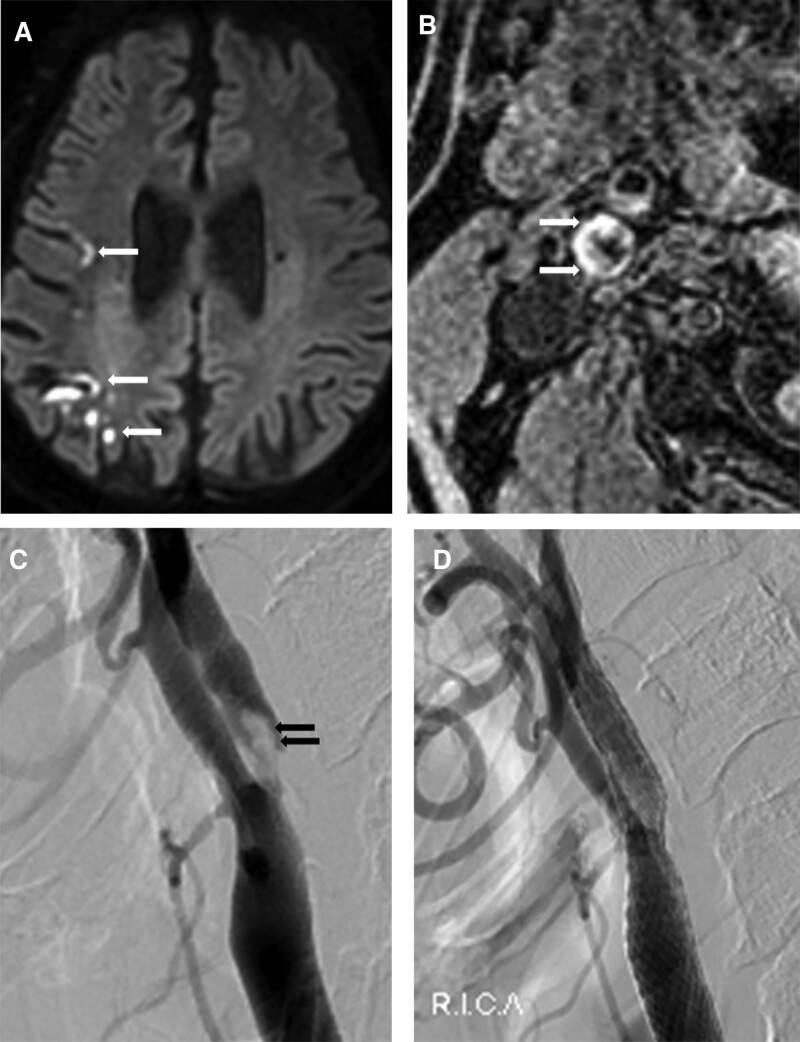
Sixty-seven-year-old man with left side weakness. (A) Diffusion-weighted imaging at admission shows a multifocal embolic infarction in the right cerebral cortex (arrows). (B) Magnetization-prepared rapid gradient-echo sequence of the right proximal carotid orifice shows a massive high signal intensity in the plaque (arrows). This high signal intensity on magnetization-prepared rapid gradient-echo sequence is indicative of intraplaque hemorrhage. (C) Right common carotid angiography shows severe stenosis with a large filling defect due to an intraluminal thrombus (arrows). (D) Right common carotid angiography after stenting using a protective device shows good patency of the stenotic lesion. An intraluminal thrombus can be seen in the protection device.

All patients underwent routine brain or stroke MRI for the evaluation of acute infarction and intracranial/extracranial stenosis or occlusion (Fig. 1A). MRI was performed using a 3.0 T scanner (Achieva; Philips Medical Systems, Best, Netherlands) equipped with a 16-channel head coil. Our protocol for carotid plaque MRI included the following 5 axial scans: TOF, black-blood T1-weighted, black-blood T2-weighted, black-blood postcontrast T1-weighted, and magnetization-prepared rapid gradient-echo (MPRAGE) sequence. The center of all sequences was at the bifurcation of the index artery with the carotid plaque. Black-blood T1-weighted, black-blood T2-weighted, and black-blood postcontrast T1-weighted sequences were obtained with a 2.0-mm slice thickness and no interslice spacing. TOF axial imaging and MPRAGE imaging were performed using a 1.0-mm slice thickness with no interslice spacing (Fig. 1B). Images were obtained with a 14 × 14-cm field of view and a matrix size of 216 × 192 pixels. The total acquisition time was approximately 30 minutes.

### 2.3. CAS procedure

All CAS procedures were performed by an interventional neuroradiologist with 15 years of experience. Systemic anticoagulation was initiated under local anesthesia via percutaneous transfemoral route with a 3000-U bolus of intravenous heparin, followed by a 1000-U/h infusion (Fig. 1C). A double-coaxial system was placed in the common carotid artery to enable stent placement. CAS was performed using the Emboshield Distal Embolic Protection System (Abbott Vascular, Plymouth, MN). Predilatation was performed using a 3-mm balloon catheter. A self-expandable stent (RX Acculinx, Plymouth, MN) was deployed (Fig. 1D). The size was chosen on the basis of the presumed parent size. Poststenting angioplasty was performed with a 5-mm-diameter balloon to achieve a residual stenosis diameter of <20%. All patients were monitored in the intensive care unit for 24 hours after the procedure.

### 2.4. Definition and outcomes

Carotid IPH was defined as the presence of a hyperintense signal within the carotid plaque that was ≥200% of the signal intensity of the adjacent neck muscle for ≤2 consecutive slices on MPRAGE images (Fig. 1B).^[16,17]^ An ulcer was defined as a depression below the plaque surface on carotid HR-MRI and carotid angiography.^[4,18]^

Symptomatic carotid artery stenosis was defined as focal neurologic symptoms and diffused weighted image (DWI)-positive imaging occurring within 1 week of CAS and attributable to an ipsilateral carotid artery vascular distribution. Positive DWI in the analysis of ipsilateral ischemic or symptomatic lesions was defined as the detection of a hyperintense signal on a DWI trace with an associated signal decrease on the apparent diffusion coefficient map.

Neurological evaluation was performed by a stroke neurologist at baseline, immediately, and 24 hours after the procedure, at the time of any change in clinical symptoms, before patient discharge and 1 month later. The evaluation consisted of the National Institutes of Health Stroke Scale (NIHSS) and modified Rankin Scale. The primary outcome measure was the incidence of procedure-related stroke, MI, or death during the 30-day periprocedural period. Procedure-related stroke was defined as an acute neurological event that lasted for ≥24 hours, which was consistent with focal cerebral ischemia. A minor stroke was defined as evidence of neurological deterioration based on a <4-point increase in the NIHSS score without the presence of aphasia, or hemianopsia, or complete recovery within 1 month. A major stroke was defined as a ≥4-point increase in the NIHSS score, presence of aphasia or hemianopsia, or any residual deficit beyond 1 month. MI was defined as the combination of elevated cardiac enzymes to a value 2 or more times the upper limit of normal, plus chest pain. The secondary outcome measures were as follows: technical success, intraluminal thrombus on initial carotid angiography, and flow arrest during stenting because of thrombus migration in the protection device; hyperperfusion syndrome, defined as the occurrence, either singly or in combination, of an ipsilateral throbbing headache with or without nausea, vomiting, ipsilateral focal seizures, or focal neurologic deficit without radiographic evidence of infarction; and intracranial hemorrhage associated with hyperperfusion syndrome or subarachnoid hemorrhage accompanied by hyperperfusion syndrome.^[16]^

### 2.5. Statistical analysis

Continuous values are expressed as means and standard deviations, while categorical data are expressed as counts and percentages. The patients were divided into IPH-positive and IPH-negative, ulcer-positive and ulcer-negative, symptomatic, and asymptomatic groups. Variables were compared among the groups using the Mann–Whitney and Fisher exact tests. Statistical significance was defined as *P* < .05. All statistical analyses were performed using R version 3.3.1 (R Foundation for Statistical Computing, Vienna, Austria).

## 3. Results

### 3.1. Patients

The baseline characteristics are summarized in Table 1. The mean patient age was 74.8 ± 7.4 years (range, 55–84). Most patients were men (n = 107, 76.4%). Sixty-seven patients (47.9%) were positive for IPH, and 53 patients (38.9%) had ulcers in their carotid plaques. Sixty-three patients (45.0%) showed acute neurological symptoms with positive DWI findings (Fig. 1A). The CAS procedure was successful in all patients. Thirteen patients had an intraluminal thrombus on the initial carotid angiography, and 12 patients showed flow arrest during stenting because of thrombus filling the protection device (Fig. 1C). No procedure-related complications were noted.

**Table 1:** Demographic data of this study.

	Patients (n = 140)
Median age, yr	74.8 ± 7.4
Age, range	55–84
Men, n (%)	107 (76.4)
Symptomatic event, n (%)	63 (45.0)
Hypertension, n (%)	98 (70.0)
Diabetes mellitus, n (%)	53 (37.9)
Hyperlipidemia, n (%)	18 (12.9)
Smoking, n (%)	28 (20.0)
Previous stroke history, n (%)	38 (27.1)
Previous cardiac disease, n (%)	27 (19.3)
IPH positive, n (%)	67 (47.9)
Ulcer positive, n (%)	53 (38.9)
Stenosis, mean	79.9 ± 9.7
Right side, n (%)	80 (57.1)
Intraluminal thrombus on initial cerebral angiography	13 (9.3)
Flow arrest during stent	12 (8.6)

IPH = intraplaque hemorrhage.

### 3.2. Outcomes based on carotid plaque and symptomatic or asymptomatic groups

The baseline data of both IPH-positive and IPH-negative patients are shown in Table 2. The incidence of intraluminal thrombi was 16.4% (n = 11) and 2.7% (n = 2) in the IPH-positive and IPH-negative groups. The incidence of intraluminal thrombi on the initial angiography was significantly higher in patients with IPH (16.4% vs 2.7%, *P* = .005). The incidence of flow arrest during CAS was 13.4% (n = 9) and 4.1% (n = 3) in the IPH-positive and IPH-negative groups, respectively. Flow arrest during CAS was significantly higher in patients with IPH than in those without (13.4% vs 4.1%, *P* = .049). The number of initial symptomatic events was significantly higher in patients with IPH than in those without (63.5% vs 35.1%, *P* < .001). Baseline data for patients with and without ulcer are shown in Table 3. The number of symptomatic events was significantly higher in the ulcer-negative group than that in the ulcer-positive group (47.15% vs 34.0%, *P* = .04). The data for symptomatic and asymptomatic patients are presented in Table 4. The incidence of intraluminal thrombi on initial angiography was significantly higher in symptomatic than in asymptomatic patients (17.5% vs 2.6%, *P* = .003). The incidence of flow arrest during CAS was significantly higher in symptomatic than in asymptomatic patients (12.7% vs 2.6%, *P* = .005).

**Table 2:** Baseline data for patients with MR-positive IPH or without IPH.

	IPH positive (n = 67)	IPH negative (n = 73)	*P*
Mean age, yrs	75.8 ± 7.0	73.8 ± 7.7	.108
Age, range	55–89	59–88	
Men, n (%)	55 (82.1)	52 (71.2)	.007
Symptomatic event, n (%)	40 (59.7)	23 (31.5)	<.001
Hypertension, n (%)	46 (68.7)	52 (71.2)	.74
Diabetes mellitus, n (%)	23 (34.3)	30 (41.1)	.41
Hyperlipidemia, n (%)	9 (13.4)	9 (12.3)	.845
Smoking, n (%)	14 (20.9)	14 (19.2)	.8
Previous stroke history, n (%)	21 (31.3)	17 (23.3)	.284
Previous cardiac disease, n (%)	12 (17.9)	15 (20.5)	.693
Angiographic data			
Stenosis, mean	80.2 ± 10.0	79.8 ± 9.2	.88
Right side, n (%)	37 (55.2)	43 (58.9)	.66
Intraluminal thrombus	11 (16.4)	2 (2.7)	.005
Flow arrest during stent	9 (13.4)	3 (4.1)	.049

IPH = intraplaque hemorrhage, MR = magnetic resonance.

**Table 3:** Baseline data for patients with ulcer-positive and ulcer-negative patients.

	Ulcer positive (n = 53)	Ulcer negative (n = 87)	*P*
Median age, yr	75.5 ± 7.8	74.3 ± 7.2	.334
Age, range	55–89	58–88	
Men, n (%)	38 (71.7)	63 (72.4)	.303
Symptomatic event, n (%)	18 (34.0)	41 (47.1)	0.04
Hypertension, n (%)	36 (67.9)	54 (62.1)	.676
Diabetes mellitus, n (%)	19 (35.8)	30 (34.4)	.702
Hyperlipidemia, n (%)	8 (15.1)	9 (10.3)	.537
Smoking, n (%)	7 (13.2)	15 (17.2)	.117
Previous stroke history, n (%)	15 (28.3)	19 (21.8)	.81
Previous cardiac disease, n (%)	12 (22.6)	16 (18.4)	.432
IPH positive	21 (39.6)	46 (52.9)	.128
Angiographic data			
Stenosis, median (IQR)	80.0 ± 9.6	80.0 ± 9.6	.822
Right side, n (%)	33 (62.3)	43 (49.4)	.339
Intraluminal thrombus	6 (11.3)	7 (8.0)	.557
Flow arrest during stent	2 (3.8)	10 (11.5)	.133

IQR = inter-quartile range, IPH = intraplaque hemorrhage.

**Table 4:** Baseline data for patients with symptomatic and asymptomatic patients.

	Symptomatic (n = 63)	Asymptomatic (n = 77)	*P*
Median age, yrs	75.0 ± 7.3	74.6 ± 7.6	.753
Age, range	58–89	55–88	
Men, n (%)	49 (77.8)	58 (75.3)	.734
Hypertension, n (%)	44 (69.8)	54 (70.1)	.97
Diabetes mellitus, n (%)	25 (39.7)	28 (36.4)	.687
Hyperlipidemia, n (%)	3 (4.8)	15 (19.5)	.001
Smoking, n (%)	16 (25.4)	12 (15.6)	.149
Previous stroke history, n (%)	22 (34.9)	16 (20.8)	.061
Previous cardiac disease, n (%)	12 (19.0_	15 (19.5)	.949
Angiographic data			
Stenosis, median (IQR)	80.3 ± 9.2	79.6 ± 9.9	.646
Right side, n (%)	33 (52.4)	47 (61.0)	.303
Intraluminal thrombus	11 (17.5)	2 (2.6))	.003
Flow arrest during stent, n (%)	8 (12.7)	2 (2.6)	.005
IPH positive, n (%)	40 (63.5)	27 (35.1)	<.001
Ulcer, n (%)	18 (28.6)	35 (45.5)	.04

IQR = inter-quartile range, IPH = intraplaque hemorrhage.

The postprocedural complications are shown in Table 5. Two patients (2.8%) with MR-positive IPH experienced a minor stroke within 30 days after CAS, and 1 patient (1.4%) with MR-positive IPH experienced MIs. One patient underwent coronary angioplasty the day after CAS and experienced nonfetal acute Q-wave MI. The number of complications in this study was small (n = 3). There was no statistically significant difference in complications (MI and minor stroke) between the IPH-positive and IPH-negative groups (4.5% vs 0%, *P* = .068). There were no significant differences in clinical symptoms after CAS or in primary outcomes, regardless of IPH, ulcer, or initial symptoms. No procedural deaths or vascular access complications occurred. No symptomatic intracranial hemorrhage was observed. Nineteen symptoms occurred in 10 patients after CAS; these symptoms included headache, nausea, vomiting, visual changes, and dizziness. All 19 patients recovered fully.

**Table 5:** Postprocedural complications after carotid artery stenting.

	Symptom			IPH			Ulcer		
	Symptomatic (n = 63)	Asymptomatic (n = 77)	*P* value	IPH(+) (n = 67)	IPH(−) (n = 73)	*P* value	Ulcer(+) (n = 53)	Ulcer(−) (n = 87)	*P* value
Death, n	0	0		0	0		0	0	
MI and stroke, n	2	1	.089	3	0	.107	2	0	.052
Headache	1	2	.575	1	2	.532	1	2	.680
Nausea	3	4	.311	2	5	.257	2	5	.465
Vomitting	3	3	.611	3	3	.548	3	3	.535
Visual change	1	1	.699	1	1	.730	1	1	.616
Dizziness	0	1	.301	0	1		0	1	.384

IPH = intraplaque hemorrhage, MI = myocardial infarction.

## 4. Discussion

This study showed that protected CAS might be feasible and safe despite the presence of unstable plaques, such as MR-positive IPH and ulceration. The rate of postprocedural complications was similar, regardless of the presence of MR-positive IPH or ulcers. Although symptomatic patients frequently have an intraluminal thrombus on initial angiography and flow arrest during CAS, these are not associated with periprocedural complications. Stenting resulted in rates of complications for all adverse events (death, stroke, or MI) that were statistically equivalent to those among patients with unstable plaques (IPH and ulceration).

Plaque vulnerability is defined as a high risk of stroke recurrence. The development of MRI has enabled noninvasive plaque imaging. Many studies have shown the accuracy of HR-MRI for the detection of unstable plaque components in carotid atherosclerosis.^[4,19–22]^ Patients with unstable features, including thin fibrous caps, IPH, ulcers, and large lipid-rich necrosis core were highly associated with a higher frequency of subsequent cerebrovascular events than those without these features.^[19,21,23]^ Unstable plaques are associated with a high incidence of distal embolism during CAS.^[6,24]^ Recent studies have attempted to determine the relationship between plaque composition and safety of CAS.^[13,14,24]^ The high-intensity signal in the plaque on 3D-TOF MRA was an independent determinant of prior ischemic strokes.^[25]^ In addition, they reported the efficacy of TOF-MRA as a screening tool for discriminating plaques at high risk of cerebral embolism during CAS.^[25]^

IPH and ulceration are risk factors in symptomatic carotid stenosis.^[19]^ In our study, 47.9% of all patients had IPH, and 38.9% had ulcers on carotid wall imaging/angiography. Some authors reported that ischemic events after CAS were more frequent in IPH-positive group and the only independent predictor of postoperative ischemic symptoms was the presence of IPH in the plaque.^[26,27]^ However, Chung et al^[24]^, who performed a similar study, reported that IPH was not a significant risk factor for cerebral embolism during protected carotid artery stent placement in patients with severe carotid stenosis. In this study, although not statistically significant, ischemic events after CAS trends to occur more in IPH positive than negative groups (2.8% vs 0%, *P* = .107).

Ulcerated carotid plaque has been established as a predictive factor for poor prognosis of carotid artery stenosis.^[6,28]^ Plaque ulceration was more common in symptomatic than in asymptomatic patients, regardless of the side of symptoms.^[18]^ Angiographic plaque surface irregularity is associated with an increased risk of ipsilateral ischemic stroke on medical treatment at all degrees of stenosis.^[7,29]^ The increase in stroke risk with degree of stenosis is partly accounted for by the parallel increase in plaque surface irregularity and thrombus formation.^[7,29]^ Several studies showed that the presence of an ulcerated plaque on preprocedural DSA increases the risk for the occurrence of DWI lesions after stenting.^[30]^ In contrast, others found that persistent ulceration after CAS improved spontaneously and did not cause embolic strokes.^[31,32]^ They explained that concerted effort to prevent persistent ulceration, such as high-pressure postdilation, is not necessary.^[31,32]^

Intraluminal thrombus on initial angiography could be considered a periprocedural complication of CAS.^[33,34]^ Coexistent thrombus of carotid plaque is associated with rupture or erosion of the lesion, and it is a feature of vulnerable plaque.^[13,24,35]^ Intraluminal thrombus is prone to dislodge from plaque, and present as a filling defect in the filter device because of guide wire passage, and balloon and stent expansion.^[36]^ Clinically, carotid stenosis with intraluminal thrombus is also associated with a poor prognosis.^[35,36]^ Intraluminal thrombi on initial angiography and flow arrest during CAS were significantly higher in patients with IPH or symptomatic patients.^[33,34]^ Patients with intraluminal thrombi showed a 7.5 times higher rate of distal embolic events than the patients without them.^[37]^ For preventing distal embolisms, protection filter devices were effective in capturing and preventing distal clot migration. ICA flow arrest due to filter occlusion during CAS is relatively common and flow arrest during CAS resolves rapidly after filter removal and does not appear to worsen outcome.^[38]^ We used an embolic protection device to prevent further complications caused by an intraluminal thrombus during the procedure. Tsumoto and Cho^[37,39]^ reported that the use of a protective device significantly decreased stroke after CAS with intraluminal thrombus. Finally considering our study, the routine use of protective devices in symptomatic patients with intraluminal thrombus appears to be effective and should receive more careful treatment during CAS placement.

Preventing distal embolisms is a major concern with CAS. Distal cerebral protection devices have been widely used during CAS, to reduce thromboembolic complications. Garg et al^[40]^ concluded that protected CAS showed a relative risk reduction of 0.59 (95% confidence interval 0.47–0.73) compared to unprotected CAS in 24 studies. The use of protection device significantly decreased stroke after CAS.^[37]^ Distal protection devices have become the standard during angioplasty and stenting for carotid artery stenosis.^[36,37,41]^

Our study had several limitations. First, this was a retrospective study; therefore, further studies are required to clarify the association between the factors and complications we investigated. Second, we categorized the symptomatic patients using DWI. The results of further research may be different from those of our study if the definition of symptomatic carotid artery stenosis changes. Third, the number of patients enrolled was too small to evaluate the association with the incidence of stroke.

## 5. Conclusion

IPH and plaque ulceration are risk factors in symptomatic carotid stenosis. However, IPH and plaque ulceration were not a significant risk factors for cerebral embolism during protected carotid artery stent placement in patients with carotid stenosis. Protected CAS might be feasible and safe despite the presence of unstable plaques, such as MR-positive IPH and ulceration. Distal protection devices have become standard device during angioplasty and stenting for carotid artery stenosis.

## Author contributions

Conceptualization: Jong-Myong Lee.

Writing – original draft: Jong-Myong Lee, Seo-Young Jeon.

Writing – review & editing: Jong-Myong Lee.
